# Pattern Recognition Receptors and DNA Repair: Starting to Put a Jigsaw Puzzle Together

**DOI:** 10.3389/fimmu.2014.00343

**Published:** 2014-07-23

**Authors:** Anton G. Kutikhin, Arseniy E. Yuzhalin, Eugene A. Tsitko, Elena B. Brusina

**Affiliations:** ^1^Laboratory for Genomic Medicine, Division of Experimental and Clinical Cardiology, Research Institute for Complex Issues of Cardiovascular Diseases under the Siberian Branch of the Russian Academy of Medical Sciences, Kemerovo, Russia; ^2^Department of Epidemiology, Kemerovo State Medical Academy, Kemerovo, Russia; ^3^Central Research Laboratory, Kemerovo State Medical Academy, Kemerovo, Russia; ^4^Department of Oncology, Cancer Research UK and Medical Research Council Oxford Institute for Radiation Oncology, University of Oxford, Oxford, UK; ^5^Department of Public Health, Kemerovo State Medical Academy, Kemerovo, Russia; ^6^Laboratory for Homeostasis Research, Division of Diagnostics of Cardiovascular Diseases, Research Institute for Complex Issues of Cardiovascular Diseases under the Siberian Branch of the Russian Academy of Medical Sciences, Kemerovo, Russia

**Keywords:** toll-like receptors, NOD-like receptors, C-type lectin receptors, RIG-I-like receptors, pattern recognition receptors, DNA repair, innate immunity, inflammation

The group of pattern recognition receptors (PRRs) includes families of toll-like receptors (TLRs), NOD-like receptors (NLRs), C-type lectin receptors (CLRs), RIG-I-like receptors (RLRs), and AIM2-like receptors (ALRs) (1–7). Conceptually, receptors constituting these families are united by two general features. Firstly, they directly recognize common antigen determinants of virtually all classes of pathogens (so-called pathogen-associated molecular patterns, PAMPs) and initiate immune response against them via specific intracellular signaling pathways (1–7). Secondly, they also recognize endogenous ligands released in cells under stress, which are known as damage-associated molecular patterns (DAMPs). Therefore, a subset of PRR-mediated immune response can be activated without an influence of infectious agents (1–7).

Long-standing data implicate that PRRs play a key role in innate and adaptive immune responses (1–7). Besides their effect on immunity, many PRRs may have a crucial impact on almost all vital cellular processes, such as cell growth, survival, apoptosis, cell cycle control, cell proliferation and differentiation, autophagy, angiogenesis, cell motility, and migration (8–14). In recent years, the evidence of the involvement of PRRs in the processes of DNA repair started to emerge. A recent comprehensive review by Harberts and Gaspari (15) has shed light on this issue; nevertheless, a number of newer investigations were performed after the publication of their paper.

One of the most investigated TLRs is TLR4, which is a transmembrane protein with an ectodomain located on the cell surface (16). The two most known TLR4 ligands are lipopolysaccharide (LPS), one of the main components of Gram-negative bacteria outer membrane, and high-mobility group protein B1 (HMGB1), which is known to be an important chromatin protein (16). It is well known that X-ray repair cross-complementing group (XRCC)5/KU80 and XRCC6/KU70 are the key non-homologous end-joining (NHEJ) repair pathway proteins (17, 18). Wang et al. observed that a diminishment of TLR4-mediated immune response may lead to reduced expression of XRCC5/KU80 and XRCC6/KU70 in mouse liver tissue and cells in response to the diethylnitrosamine, therefore, being a cause of the DNA repair impairment and reactive oxygen species (ROS) accumulation (17, 18). However, when TLR4^−/−^ mice and wild-type mice were locally exposed to ultraviolet B (UVB, shortwave radiation), the expression of DNA repair gene xeroderma pigmentosum, complementation group A (*XPA*), and production of interleukins (ILs) 12 and 23 were significantly higher (19). Further, cyclobutane pyrimidine dimers were repaired more efficiently in the skin and bone marrow-derived dendritic cells (DCs) of TLR4^−/−^ mice (19). The addition of anti-IL-12 and anti-IL-23 antibodies to bone marrow-derived DCs of TLR4^−/−^ mice before UVB exposure inhibited repair of cyclobutane pyrimidine dimers along with a decline of *XPA* gene expression; similarly, the addition of TLR4 agonist to wild-type bone marrow-derived DCs lowered *XPA* gene expression and diminished repair of cyclobutane pyrimidine dimers (19). Hence, the activation of TLR4 signaling by ultraviolet radiation may launch a specific pathway and result in decrease of IL-12 and/or IL-23 production, thereby reducing the expression of genes encoding DNA repair enzyme such as *XPA* (19). According to these studies (17–19), TLR4 may both upregulate and downregulate distinct DNA repair proteins, and possibly does it in different ways in distinct cell types, so its exact role in DNA repair remains unclear.

Certain TLRs are located on the endoplasmic reticulum membrane (in a resting state) or on the endosomal/lysosomal membrane (upon ligand stimulation and trafficking) (20). Among these are TLR7, TLR8, and TLR9 (20). The main ligands for TLR7 and TLR8 are imidazoquinolines, ssRNA, and antiphospholipid antibodies, while the main ligands for TLR9 are bacterial and viral CpG DNA and IgG-chromatin complexes (20). However, all these receptors signal via the protein encoded by myeloid differentiation primary response gene 88 (*MyD88*) (20). Tsukamoto et al. found that 8-mercaptoguanosine (8SGuo) induces the activation-induced cytidine deaminase (AID) expression and double-strand breaks (DSBs) through TLR7–MyD88-dependent pathway in cluster of differentiation (CD)38- or B cell receptor (BCR)-activated B cells (21). Nevertheless, imiquimod, a TLR7/8 agonist, which is used in the treatment of certain non-melanoma skin cancer, increased an expression and nuclear localization of *XPA* gene and other DNA repair genes in a MyD88-dependent manner (22). In addition, as it was detected by Fishelevich et al. imiquimod enhanced DNA repair and accelerated the resolution of cyclobutane pyrimidine dimers after an exposure of bone marrow-derived cells to ultraviolet light (22). Imiquimod-activated cutaneous antigen presenting cells were characterized by better DNA repair in comparison with resting antigen presenting cells under the exposure to both non-ionizing and ionizing radiation (22). Moreover, topical application of imiquimod before the exposure to ultraviolet light had a protective effect and reduced the number of cyclobutane pyrimidine dimers-positive antigen presenting cells (22). Therefore, the role of TLR7 and TLR8 in DNA repair may differ depending on their influence on the specific DNA repair proteins or on the cell type, as in the case with TLR4.

In the study of Zheng et al., TLR9-stimulated CD4 T cells demonstrated an increased capacity to repair ionizing radiation-induced DSBs, whereas the treatment of irradiated CD4 T cells with TLR9 ligands along with checkpoint kinase (Chk)1/2 inhibitors or along with ataxia telangiectasia mutated/ataxia telangiectasia and Rad3 related (ATM/ATR) inhibitor wortmannin abrogated the improvement of DNA repair observed previously (23). In addition, TLR9 stimulation did not elevate DNA repair rates after an exposure to ionizing radiation in TLR9^−/−^ and MyD88^−/−^ CD4 T cells; thus, TLR9-induced DNA repair may be mediated by Chk1/2 and ATM/ATR via MyD88-dependent pathway (23). Klaschik et al. performed a global gene expression analysis on mouse splenic cells and revealed that CpG DNA, a ligand for TLR9, may cause the activation of genes responsible for DNA repair 3–5 days after an intraperitoneal injection, so the long-term enhancement of DNA repair after TLR9 stimulation is possible (24). Sommariva et al. carried out an *in silico* analysis of DNA repair genes in data sets obtained from murine colon carcinoma cells in mice injected intratumorally with synthetic oligodeoxynucleotides expressing CpG motifs (CpG–ODN, a TLR9 agonist) and from splenocytes in mice treated intraperitoneally with CpG–ODN (25). According to their results, CpG–ODN downregulated DNA repair genes in tumors, but upregulated them in immune cells (25). Moreover, «CpG-like» expression pattern of CpG–ODN modulated DNA repair genes was associated with a better outcome of patients with breast and ovarian cancer treated by DNA-damaging agents than «CpG-untreated-like» expression pattern, so these genes may determine tumor cell response to genotoxic drugs (25). It seems to be that the exact role of TLR9 in DNA repair substantially depends on the cell type.

It was found that MyD88 mediates the optimal activation of the Ras/mitogen-activated protein kinase (MAPK) pathway by binding to extracellular signal-regulated kinase (ERK) and protecting it from dephosphorylation (26–29). In accordance with the data obtained by Kfoury et al., MyD88 inhibition may lead to defective excision repair cross-complementing rodent repair deficiency, complementation group 1 (ERCC1)-dependent DNA repair and to accumulation of DNA damage (29, 30). In addition, abrogation of *MyD88* gene expression sensitizes cancer cells to genotoxic agents such as platinum salts *in vitro* and *in vivo* (29, 30). It is worthy of note that platinum-based chemotherapeutic agents (cisplatin, carboplatin, and oxaliplatin) cause DNA damage that is preferentially repaired by the nucleotide excision repair (NER) pathway, which is implicated in the repair of DNA single-strand breaks (SSBs), and ERCC1 predominantly functions as NER enzyme via Ras-MAPK pathway (29, 30). So, MyD88-dependent Ras-MAPK-mediated activation of ERCC1 may play a major role in DNA repair (29, 30). However, Lai and Egan reported that early induction of DSBs in mouse colonic epithelial cells by ionizing radiation was independent of the presence and absence of *MyD88* gene expression (31). Notwithstanding, they observed a later loss of DSBs and an enhanced activation of DSB repair pathways in MyD88^−/−^ mice compared to control mice (31). It seems to be that MyD88 has no specific inhibitory effects regarding the pathways of DSB repair since both the NHEJ and homologous recombination (HR) repair pathways were over-activated in the absence of MyD88 (31). Possibly, MyD88-mediated signaling pathway may regulate the repair of SSBs and DSBs in a distinct way via activation or inhibition of the proteins specific for each of pathways responsible for the repair of SSBs and DSBs.

The only study investigating the role of NLRs in DNA repair was carried out by Licandro et al. regarding NLR family, pyrin domain containing 3 (*Nlrp3*) gene (32). The ectodomain of NLRP3 recognizes certain DAMPs that may lead to the assembly of inflammasome and, hence, to the development of aseptic inflammation (33). The authors exposed murine DCs to monosodium urate, rotenone, and γ-radiation, and detected a lesser level of DNA fragmentation in Nlrp3^−/−^ DCs compared to wild-type DCs (32). Moreover, Nlrp3^−/−^ DCs experienced significantly less ROS-mediated DNA damage, and a significantly lower expression of several genes involved in DSB and base excision repair (BER) was revealed in wild-type DCs (32). These genes included *XRCC1, RAD51*, 8-oxoguanine–DNA glycosylase 1 (*OGG1*), breast cancer 1, early onset (*BRCA1*), DNA polymerase beta (*POLB*), and thymidylate synthase (*TYMS*) (32). It was demonstrated that DSB and BER enzymes responsible for repair of 8-oxoguanine, which is a DNA adduct formed as a result of oxidation, and therefore, is considered a marker of oxidative stress, were more active in Nlrp3^−/−^ cells in comparison with wild-type DCs (32). In addition, Nijmegen breakage syndrome 1 (NBS1), another protein involved in DNA repair, was highly phosphorylated in Nlrp3^−/−^ DCs compared with wild-type DCs, indicating greater efficacy of DNA repair in the absence of *Nlrp3* gene expression (32).

Taken together, these reports strongly implicate PRRs, in particular TLRs (TLR4, TLR7, TLR8, and TLR9) and NLRs (NLRP3), as major regulators of DNA repair (Table S1 in Supplementary Material). According to the above-mentioned findings, these five receptors may affect the expression of at least eight enzymes (XRCC1, XRCC5, XRCC6, XPA, BRCA1, POLB, TYMS, OGG1, and RAD51) and two ILs (IL-12 and IL-23) involved in various mechanisms of DNA repair. Further, PRRs are responsible not only for the initiation of one specific DNA repair pathway, but a number of such pathways repairing different types of DNA damage, i.e., oxidation, alkylation, and hydrolysis of bases, bulky adducts, SSBs, DSBs, and crosslinks. Interestingly, the effect of PRRs on DNA repair may vary between cell types and cell lines, which address a number of questions to be answered in future studies.

Nowadays, we are only beginning to put the pieces of this puzzle together. Current vision of this topic is blurred, although a preliminary picture based on recent research can be drawn (Figure 1). Both TLRs located on the cell surface and thus responsible for the recognition of the pathogen envelope molecular patterns (TLR4) and TLRs located on the endoplasmic reticulum, endosomal, or lysosomal membrane, and therefore, responsible for the recognition of pathogen nucleic acids (TLR7, TLR8, and TLR9) are involved in DNA repair. Therefore, other TLRs belonging to any of these groups may also participate in such processes. Definitely, the cytokine-mediated DNA repair feedback loop is not restricted to IL-12 and IL-23, and might consist of much greater number of cytokines, possibly TLR-regulated cytokines [IL-1, IL-2, IL-6, IL-8, IL-10, IL-13, IL-27, macrophage inflammatory protein-1 (MIP-1), monocyte chemotactic protein-1 (MCP-1), regulated on activation, normal T-cell expressed and secreted (RANTES), suppressor of cytokine signaling (SOCS), granulocyte-macrophage colony-stimulating factor (GM-CSF), tumor necrosis factor-α (TNF-α), interferon (IFN)-α, IFN-β, IFN-γ, and IFN-inducible proteins]. Furthermore, the exact composition of the growth factors-mediated DNA repair signaling pathway is still elusive; importantly, this pathway may have a particular importance since it includes both MyD88 and Ras-MAPK pathways, representing an interesting example of a crosstalk between canonical TLR MyD88-mediated signaling pathway and Ras-MAPK signaling pathway. In addition, there are no studies on the feasible influence of CLRs, RLRs, and ALRs on DNA repair. The improvement of our understanding of the role of PRRs in DNA repair may find implications for clinical medicine; peculiarities of PRRs functioning should definitely be considered when assessing the possibility of the use of PRR agonists in therapy of various diseases such as cancer. No doubt, further in-depth investigations are needed for deciphering the role of PRRs in sophisticated mechanisms of DNA repair.

**Figure 1:**
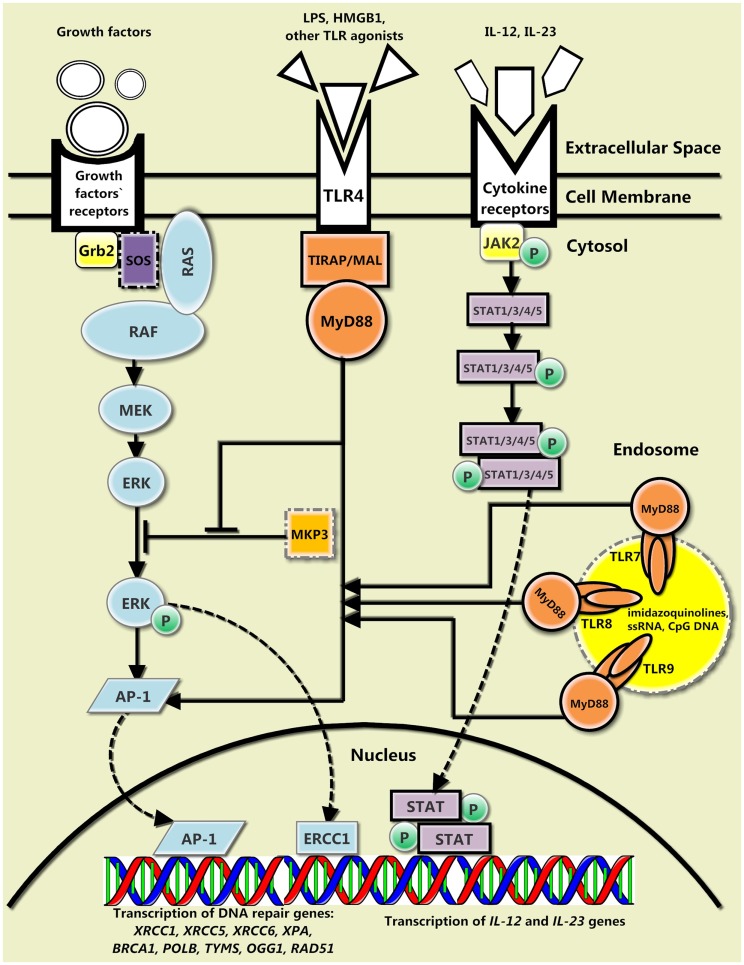
**The general interplay between the canonical TLR signaling pathway, the cytokine-mediated DNA repair feedback loop, and the growth factors-mediated signaling pathway**. There are three main TLR-mediated pathways of DNA repair. The protein encoded by myeloid differentiation primary response gene 88 (MyD88) and its downstream signaling proteins (not shown) may inhibit mitogen-activated protein kinase phosphatase 3 (MKP3), which hinders phosphorylation of extracellular signal-regulated kinase (ERK), and therefore, prevents further signaling via Ras-MAPK pathway. In addition, MyD88 and its downstream signaling proteins (not shown) along with pERK activate AP-1 transcription factor, which promotes transcription of certain DNA repair genes. Finally, IL-12 and IL-23, which enhance DNA repair and whose transcription is also amplified by MyD88-regulated transcription factors, bind to their receptors, activate Janus kinase-signal transducer and activator of transcription (JAK-STAT) signaling pathway, and increase further transcription of their own encoding genes.

## Conflict of Interest Statement

The authors declare that the research was conducted in the absence of any commercial or financial relationships that could be construed as a potential conflict of interest.

## Supplementary Material

The Supplementary Material for this article can be found online at http://www.frontiersin.org/Journal/10.3389/fimmu.2014.00343/abstract
