# Supplementary material for: Pattern Recognition Receptors and DNA Repair: Starting to Put a Jigsaw Puzzle Together
Source: Front Immunol. 2014 Jul 23;5:343. doi: 10.3389/fimmu.2014.00343 (PMC4107940; doi:10.3389/fimmu.2014.00343)
Supplement: Supplementary file 1 [file Table_1.PDF]

Table S1 | Pattern recognition receptors and the DNA repair components that are regulated by them.

Pattern recognition receptor (PRR) or protein of the PRR-mediated pathway	Modulation of the PRR-mediated immune response	DNA repair enzyme	DNA repair pathway	Reference
TLR4	Diminishment	Reduced expression of XRCC5/KU80 and XRCC6/KU70	NHEJ	(17, 18)
	Diminishment	Increased expression of XPA	NER	(19)
TLR7 and TLR8	Stimulation (use of agonists)	Induction of AID expression and DSBs	Various	(21)
	Stimulation (use of agonists)	Increased expression of XPA	NER	(22)
TLR9	Stimulation	Activation of Chk1/2, ATM/ATR (DNA repair via the cell cycle control) Greatly depends on the cell type (opposite effects in tumor or immune cells)	Various	(23–25)
MyD88	Inhibition	Deficiency of ERCC1-mediated DNA repair	NER	(30)
	Inhibition	Activation of various DNA repair enzymes	NHEJ, HR	(31)
NLRP3	Diminishment	Increased expression of XRCC1, RAD51, OGG1, BRCA1, POLB, TYMS, and NBS1	BER, DSB repair	(32)
